# A Commentary on the Use of Bivalve Mollusks in Monitoring Metal Pollution Levels

**DOI:** 10.3390/ijerph18073386

**Published:** 2021-03-25

**Authors:** Chee Kong Yap, Moslem Sharifinia, Wan Hee Cheng, Salman Abdo Al-Shami, Koe Wei Wong, Khalid Awadh Al-Mutairi

**Affiliations:** 1 Department of Biology, Faculty of Science, Universiti Putra Malaysia, Serdang 43400, Selangor, Malaysia; wongkoewei@gmail.com; 2 Shrimp Research Center, Iranian Fisheries Science Research Institute, Agricultural Research, Education and Extension Organization (AREEO), Bushehr 75169-89177, Iran; moslem.sharifinia@yahoo.com; 3 Faculty of Health and Life Sciences, Inti International University, Persiaran Perdana BBN, Nilai 71800, Negeri Sembilan, Malaysia; wanhee.cheng@newinti.edu.my; 4 Indian River Research and Education Center, IFAS, University of Florida, Fort Pierce, FL 34945, USA; alshami200@gmail.com; 5 Department of Biology, Faculty of Science, University of Tabuk, P.O. Box 741, Tabuk 71491, Saudi Arabia; kmutairi@ut.edu.sa

**Keywords:** bivalves, shells, monitoring, chemical pollutants

## Abstract

The objective of this commentary is to promote the use of bivalves as biomonitors, which is a part of the continual efforts of the International Mussel Watch. This commentary is an additional discussion on “Bivalve mollusks in metal pollution studies: From bioaccumulation to biomonitoring” by Zuykov et al., published in Chemosphere 93, 201–208. The present discussion can serve as a platform for further insights to provide new thoughts and novel ideas on how to make better use of bivalves in biomonitoring studies. The certainty of better and more extensive applications of mollusks in environmental monitoring in the future is almost confirmed but more studies are urgently needed. With all the reported studies using bivalves as biomonitors of heavy metal pollution, the effectiveness of using Mussel Watch is beyond any reasonable doubts. The challenge is the development of more accurate methodologies for of heavy metal data interpretation, and the precision of the biomonitoring studies using bivalves as biomonitors, whether in coastal or freshwater ecosystems. Lastly, inclusion of human health risk assessment of heavy metals in commercial bivalves would make the research papers of high public interest.

## 1. Introduction

Firstly, the well-written review paper published by Zuykov et al. [1] promoting the use of bivalves as biomonitors is focused on and discussed in the present commentary paper. As of January 2021, this paper had been cited by 165 papers based on Google Scholar. This monitoring paper using bivalves is a part of a continual effort of the International Mussel Watch. This mussel monitoring work should have been highly commended and supported from its inception when it was suggested by Goldberg [2] and should continue to be for educational purposes. This is because of the fact that the pollutant levels in bivalves will greatly affect human health.

We must say that it is not our intention to offend the good researchers in the paper by Zuykov et al. [1], but our honest comments are based on science. As researchers working on biomonitoring studies using mussels since 1998, we want to humbly comment and add more discussion on the above review paper, based on the following highlighted points of view as presented the paper.

1. The uses of bivalves as biomonitors of metal pollution are primitive methods.
2. The use of shells to construct pollution history blueprint is futile.
3. There has not been any documented evidence of severe health effects of bivalves due to metal accumulation.

## 2. Comments and Discussions

Two of the specific goals of the paper by Zuykov et al. [1] were to (1) “discuss the biomonitoring of metal pollution using bivalves”, soft tissues and shells as a mean of environmental “health monitoring”, and (2) “reveal additional information of metal pollution in aquatic environments based on the observation of the internal shell surface through scanning electron microscope”. However, these objectives were not answered and not actually summarized or briefly discussed in the abstract.

### 2.1. The Uses of Bivalves as Biomonitors of Metal Pollution Are Primitive Methods

It seems to the general readers that the biomonitoring of metals using bivalves is not effective and is an old research method. We disagree with this highlighted point because there has been an influx of reported studies, before, now and expectedly in the future too, using bivalves for pollution studies. As reviewed in Table 1, many such studies can be found in different countries up until January 2021. In our view, we would not say “primitive” since this could totally discourage the use of bivalves in metal pollution monitoring and funding for such studies would not be continued. We think, besides bioaccumulation data, the effectiveness of using identified bivalves for the biomonitoring of metal pollution for spatial distribution and comparison should be improved upon and the accuracy enhanced, in future studies.

In fact, Zuykov et al. [1] made a substantial, careful and excellent review on the use of bivalve soft tissues and shells in biomonitoring studies from numerous papers. Even similar early papers by Boening [3], Yap et al. [4] and Yap [5] have given suggestions for biomonitoring assays using marine mussels. This trend continues in recent papers such as Nędzarek et al. [6] and Wepener and Degger [7]. All of this clearly shows the potential of mollusks as sentinel organisms of heavy metal pollution for future studies. In particular, Yap [5] and Yap et al. [8] proposed that the recommended criteria for marine mussels could be applied to other mollusk species, establishing them as good biomonitors of heavy metal pollution. None of these papers mentioned that “the biomonitoring work is not far advanced”. Therefore, in our opinion, the uses of bivalves as biomonitors of metal pollution are far reaching methods instead of being primitive.

The paper entitled “From bioaccumulation to biomonitoring” is somewhat confusing to the readers too. However, when the abstract is carefully looked into, “biomonitoring” means “estimation of environmental quality”. In fact, biomonitoring is better explained as “regular and systematic use of living organisms to evaluate changes in environmental or water quality that involves repetitive measurements of pollutants/chemicals”, which is central to aquatic toxicology and ecotoxicology. In the science of analytical chemistry, biomonitoring is the estimation of the body burden of poisonous synthetic compounds, elements, or their metabolites in bioorganic substances [98].

Before we can better estimate the environmental quality, we think the current challenges now are to enhance the precision of the biomonitoring studies using bivalves as biomonitors, whether in coastal or freshwater ecosystems. These challenges have been little discussed in the recent literature (Table 1). The metal bioaccumulation data in bivalves could be influenced by several abiotic and biotic factors. For example, the abiotic (such as pH, salinity, etc.) and the biotic (sizes, gender, genetic differentiation, predation, competition) factors could simply alter the metal bioaccumulation data in the body tissues of the bivalves [99].

Based on the title of the paper again, most ecotoxicologists will understand that the use of bivalves for biomonitoring purposes is of high novelty. However, bivalves have been widely employed and recognized as good biomonitors of four major collective pollutants—namely, halogenated hydrocarbons, transuranics, heavy metals and petroleum, as mentioned in the Mussel Watch Program [2]. This is because mussels have many of the important characteristics of good biomonitors [100,101] of coastal pollution. According to Farrington et al. [101], bivalves are widely distributed in coastal waters across the globe, are sessile in nature, are capable of accumulating pollutants at high concentrations (by factors of 10^3^ to 10^5^), seem to be unaffected by pollutants, are important seafood products heavily consumed in certain parts of the world and therefore can be a risk to human health. For *Perna viridis* particularly, extensive studies had been reported from the Asia-Pacific coastal regions [102,103]. From at least 65 of the high impact publications as seen in Table 1, four patterns can be deduced.

Firstly, publications on metal studies in mussels have been consistently and widely accepted worldwide in more than 50 countries around the world’s coastal regions, as summarized in Table 1, spanning from the 1970s until January 2021.

Secondly, there have been continuous efforts made and research grant allocations given to study heavy metal levels in bivalve mollusks in both marine and freshwater environments, but mainly in marine mussels, ever since the introduction of the famous International Mussel Watch, initially proposed by Prof. Goldberg [2]. This shows that metal pollution studies using mussels are not only truly scientific research studies based on biomonitoring, but they have also triggered other newly emerging scientific studies and topics.

Thirdly, the summary in Table 1 highlights that biomonitoring studies using mussels occur in developed, developing and underdeveloped countries all around the world. This greatly signifies that Mussel Watch is a cost-effective study and the idea has spread in a very positive and fruitful manner in all coastal areas around the world. The benefits include developing expertise in the areas of ecotoxicology, biology and environmental sciences, which eventually lead to opportunities for training postgraduate research students.

Fourthly, the common heavy metals such as Hg, Cd, Cu, Pb and Zn have always been focused upon with Ag, Al, As, Ba, Ni, Co, Cr, Fe, Mn, Se, Sr, Sn, Ti and V. In fact, the biomonitoring studies using bivalves have been expanded to more metals or elements including rare earth elements (Table 1). Therefore, if biomonitoring studies using mussels are “not far advanced”, then this work is a futuristic study that is influential in many regions around the world with its ever-expanding trends, although it is traditional in its origins and ideas.

### 2.2. The Use of Shells to Construct Pollution History Blueprint Is Futile

We have conducted a comprehensive review covering papers published up until January 2021 on the use of bivalves’ shells for the biomonitoring of metals. In addition, based on the review presented in Table 2, more related studies give evidence that such studies are continuing now and in the future. The main reason for this is because shells have potential for biomonitoring metal pollution, and it is believed that they can be reconstructed to reflect the metal pollution history. This challenge remains.

The original idea of using the shell as a reliable biomonitoring material for the reconstruction of pollution history (see [133,134,135,136,137,138]) is acceptable and is believed to be workable and reliable when compared to the use of soft tissues. This is the main reason why so many researchers conducted such related studies using molluscs’ shells for the biomonitoring of metal pollution, to compare the current with the past history of metal bioaccumulation in bivalve shells as reviewed in Table 3. The reason why we compared the previously collected shells with the current ones is because we can logically establish the past pollution history as shells can be easily stored without having to be kept in a freezer (Table 3).

Overall, researchers are trying to provide more data and evidence, but the reconstruction of pollution history using shells remains a big challenge. Therefore, while we wait for more evidence to prove the positive usage of mollusc shells for elucidating pollution history, we think it is premature to say “Shells cannot be reliably used for the reconstruction of the pollution history”. In fact, almost all the papers reviewed by Zuykov et al. [1] concluded on the positive use of bivalves’ shells for biomonitoring of metal pollution and none stated the contrary. Rather, we think more studies are needed to establish the use of bivalves’ shells as good biomonitors and to reconstruct the pollution history of heavy metals.

### 2.3. There Has Not Been Any Documented Evidence of Severe Health Effects of Bivalves Due to Metal Accumulation

If the paper is only based on the reviewed literature, we disagree with the statement that “There are no effects of high metal bioaccumulation on the health status of bivalves”. In fact, a number of biomarkers have been used to show the health effects due to metal bioaccumulation in the mussels and these have also been evidently proven (Table 2). For example, the condition index (CI) of bivalves has been widely used as a health status measure in response to high metal bioaccumulation in bivalves due to pollution effects [139]. Unless there are other major factors, the metal levels in the body tissues and the CI of bivalves are negatively correlated. Based on their studies, de los Ríos et al. [140] reported the detrimental effects on mussels’ health from metals and xenoestrogenic endocrine disruptors found in some discharges. Signa et al. [119] reported that a lower condition index and phospholipids, as well as higher total and neutral lipids in mussels from Augusta, indicated the occurrence of physiological and biochemical stress responses to metal exposure and accumulation.

Riveros et al. [141] measured cellular biomarkers (the levels of vacuole formation and the amount of lipofuscin granules in the digestive system as well as lysosomal stability in haemocytes) in the mussel *Perumytilus purpuratus* from the intertidal zones of San Jorge Bay, Antofagasta, Chile. Al-Subiai et al. [143] concluded based on the multibiomarker approach using *Mytilus edulis* that a visible relationship between genotoxicity and higher-level effects was present and this could be used to determine various short- and long-term toxic effects of Cu. Marigomez et al. [144] reported that biomarkers in depicted site-specific profiles served as essential diagnostic tools for health assessments of metal pollution not just of mussels but of the marine ecosystem as well. Their study supported a combined use of both caged and native mussels in highly polluted areas to monitor the biological effects of pollution in mussels through the integrative biomarker approach. Brooks et al. [150] highlighted the biological effects of pollutants on the blue mussel (*Mytilus* spp.) to evaluate the effluents discharged from the Sydvaranger mines. The results of the integrated biological responses were in line with the source of pollution judging by the distance and location between the mussels and the discharge outlet and the expected exposure to the mine effluents. González-Fernández et al. [151] concluded that the vast variability of the population of the mussel, *Mytilus galloprovincialis*, caused by environmental factors such as food availability in a certain monitoring program might conceal the effects of pollutants on the biomarkers. Chandurvelan et al. [152] reported that the specific tissue or the whole organism response of *Perna canaliculus* towards metal pollutants reveals crucial information on the biological stress responses, denoting the importance of such measurements in biomonitoring programs in New Zealand. Giarratano et al. [147] reported that the gill of the mussel *Aulacomya atra* is an actively proliferating tissue and is the first target of contaminants (Fe, Al, Zn, Cu, Cd and Pb) present in the water, so that changes in its antioxidant system can provide an earlier warning signal than changes in other tissues. Lekube et al. [148] concluded that cellular biomarkers in *Mytilus galloprovincialis* were extremely sensitive and quick to respond to changes in environmental pollutants such as heavy metals. The results obtained by Tsangaris et al. [62] confirmed the usefulness of the integration of biological effect measurements and chemical analysis in *Mytilus galloprovincialis* for the assessment of chemical contamination including those by Cu, Ni, Fe and Zn in coastal waters. Other studies using biomarkers as evidence to show the effects of accumulated metals in relation to the health of the mussels investigated are shown in Table 3.

According to Lam and Gray [175], biomarkers can be used for the quantitative determinations of physiological and biological changes that are observed in cells, body fluids, tissues or organs of an organism. These serve as indicators of exposure to xenobiotics and their effects. Therefore, the accumulated metals on the health of bivalves can be reflected by looking into the biomarkers of the mussels.

## 3. Human Health Risk Assessment of Heavy Metals in the Bivalves

Table 4 shows a review on the human health risk assessments of heavy metals in bivalves from some available literature. With the increasing trends of anthropogenic inputs into aquatic ecosystems, the commercial shellfish from these areas are of much public concern. Therefore, from Mussel Watch monitoring to human health risk assessment of heavy metals are of high significance [176,177]. It seems that with heavy metal data in the bivalves without the human health risk assessment of the heavy metals would make the whole research finding of low novelty.

## 4. Conclusions

With all the reported studies using bivalves as biomonitors of heavy metal pollution, the effectiveness of using Mussel Watch is beyond any reasonable doubts. The challenge is on the development of more accurate methodology of heavy metal data interpretation and the precision of the biomonitoring studies using bivalves as biomonitors, whether in coastal or freshwater ecosystems. Such ideas have been marginally proposed in the literature [189,190]. In addition, human health risk assessment of heavy metals in commercial bivalves will be of much public interest. Therefore, inclusion of consumer perspectives on the heavy metal data is of high importance. Lastly, the Mussel Watch approach could be proposed as Crop Watch in studies of ecotoxicological genetics [191]. 

This commentary is an additional discussion on “Bivalve mollusks in metal pollution studies: From bioaccumulation to biomonitoring” by Zuykov et al. [1]. It is hoped that this communication paper will serve as a platform for further discussion that can provide new thoughts and novel ideas on how to make better use of bivalves, both marine and freshwater, in biomonitoring studies. It is certain that more future studies using bivalve mollusks as biomonitors of pollution are much needed.

## Figures and Tables

**Table 1:** A review on the use of bivalves’ soft tissues for metal pollution studies from some of the available literature.

No.	Countries (year)	Species	Metals Investigated	References
1	Scotish coastal waters, Scotland (17 sites; unspecified)	*Mytilus edulis*	Cu, Zn, Cd and Pb	[9]
2	The northern part of Port Phillip Bay, Victoria, Australia (3 sites; 1974–1975)	*Mytilus edulis*	Zn, Cd, Pb, Cu, Fe and Mn.	[10,11]
3	Northern Ireland (11 sites; 1980–1981)	*Mytilus edulis*	Cu, Cd, Zn, Pb, Hg, Cr and Ni	[12]
4	The Gulf of Trieste, Italy (4 sites; 1974–1984)	*Mytilus galloprovincialis*	Co, Ni, Co, Cd, Hg and Pb	[13]
5	Long Island Sound (10 sites; 1983)	*Mytilus edulis*	Cd and Cu	[14]
6	Southwest Iceland (48 sites; 1978)	*Mytilus edulis*	Hg, Cd, Pb, Cu and Zn	[15]
7	Coastal North Sea and the Estuaries of Ems, Western and Eastern Scheldt (The Netherlands) (9 sites; 1979–1983)	*Mytilus edulis*	Hg, Pb, Cd, Cu and Zn	[16]
8	The Gulf of Thailand, Thailand	*Perna viridis*	Zn, Mn, Cu, Cr, Ni and Cd	[17]
9	Chilean coasts (8 sites; 1992)	*Perumytilus purpuratus*, *Semelle solida* and *Tagelus dombeii*	Cd, Cu and Zn	[18]
10	Southeast Alaska, USA (4 sites; 1981–1982)	*Mytilus edulis*	As, Cu, Zn, Cd, Mo, Pb, and Cr	[19]
11	Bergen Harbor Area, Western Norway (Norway) (20 sites; 1993)	*Mytilus edulis*	Zn, Cu, Pb, Cd and Hg,	[20]
12	The Gulf of Aden, Yemen	*Perna perna*	Cd, Pb, Zn, Cu, Mn, and Fe	[21]
13	Mazatlan Harbour, Mexico (3 sites; 1996)	*Mytella strigata*	Cd, Pb, Zn, Cu, Ag, Cr, Co, Ni, Mn, and Fe	[22]
14	Taiwan coastal waters, Taiwan (5 sites; 1991–1996)	*Crassostrea gigas*; *Meretrix lusoria*	Cu, Zn, Pb, Cd, As and Hg	[23]
15	Kyushu Island, Japan (3 sites; 1994)	*Mytilus edulis*	Hg, Ag, Cr, Co and Ni	[24]
16	Danube Delta, Romania (12 lakes; 1994–1995)	*Anodonta anatina* *, Unio pictorum, U. tumidus*	Ag, As, Cd, Co, Cu, Cr, Ni, Pb, Se and Zn	[25]
17	Agadir Marine Bay, South of Morocco (2 sites; 1994)	*Mytilus galloprovincialis*; *Perna perna*	Cd, Cu and Zn	[26]
18	The Gulf of Maine, USA (56 sites; 1991–1997)	*Mytilus edulis*	Ag, Al, Cd. Cr, Cu, Fe Hg, Ni, Pb and Zn	[27]
19	Southern Baltic, Poland (23 sites; 1997)	*Mytilus edulis trossulus*	Hg, Cd, Pb, Ag, Cu, Zn, Cr, Ni, Co, Mn, and Fe	[28]
20	Venezuala and Trinidad (8 sites; 1999)	*Perna viridis*	Cd, Cu, Cr, Hg, Ni and Zn	[29]
21	Island of Murano (Venice, Italy)(4 sites; 1999)	*Mytilus galloprovincialis*	Fe, Mn, Zn, Cu, Cr, Pb, Ni, Ag and As	[30]
22	Peninsular Malaysia coasts (20 sites; 1997–2001)	*Perna viridis*	Cd, Cu, Pb and Zn	[31]
23	Hong Kong (2 sites; unspecified)	*Perna viridis*	Cu, Co, Ni, Cd, Zn, Mn, Cr, Fe and Pb	[32]
24	Korea (7 sites; 1998–1999)	*Mytilus galloprovincialis*	Cd, Co, Cu, Cr, Fe, Hg, Mn, Ni, Pb, Sn, Ti and Zn	[33]
25	Singapore (8 sites; 2002)	*Perna viridis*	As, Cd, Cr, Cu, Ni, Pb and Zn	[34]
26	East coast of China (7 sites; 2001)	*Perna viridis*; *Mytilus edulis*	Ag, As, Cd, Cr, Ni, Pb, Se, Zn, Cu, Fe and Hg	[35]
27	The Gulf of Gdansk, Baltic Sea, Poland (5 sites; 2000–2001)	*Mytilus trossulus*	Cu, Zn, Cd, Fe, Pb, Mn and Ni	[36]
28	Sea of Okhotsk and the Sea of Japan (4 sites; 2001)	*Crenomytilus grayanus*	Zn, Fe, Ni, Cu, Mn, Cd, and Pb	[37]
29	Duy Minh and An Thin, northern part of Vietnam (2 sites; 2001)	*Pletholophus swinhoei*	As, Ba, Be, Ca, Cd, Cr, Cu, Fe, K, Mn, Ni, P, Pb, Rb, S, Se, Sr, Ti and Zn	[38]
30	Hong Kong coastal waters (5 sites; 1998–2003)	*Perna viridis*	Al, As, Cd, Cr, Cu, Fe, Hg, Mn, Ni, Pb, Zn and V	[39]
31	Western Scheldt estuary (The Netherlands) (4 sites; 1996–2002)	*Mytilus edulis*	Cd, Co, Cr, Cu, Fe, Mn, Ni, Pb and Zn	[40]
32	Karnataka, Southwest Coast of India (28 sites; 2002)	*Perna viridis*	Cd, Cr, Cu, Fe, Mn, Ni, Pb, and Zn	[41]
33	Western coast of Senegal (1 site; 2002–2003).	*Perna perna*; *Crassostrea gasar*	Cd, Cu and Zn	[42]
34	Göta Älv Estuary (SW Sweden) (5 sites; 2002–2003)	*Mytilus edulis*	Cd, Cu, Hg, Pb and Zn	[43]
35	Taranto Gulf, Ionian Sea, Southern Italy (2 sites; 1999–2000).	*Mytilus galloprovincialis*	Cd, Cu, Pb, Zn, Fe and As	[44]
36	Galicia and Gulf of Biscay (Spain) (6 sites; 2000–2004)	*Mytilus galloprovincialis*	Cd, Hg, Pb, Cu and Zn	[44]
37	Hong Kong coastal waters (5 sites; 2004–2005)	*Perna viridis*	Cd, Cr, Pb, Cu and Zn	[45]
38	Eastern Black Sea, Turkey (5 sites; unspecified)	*Mytilus galloprovincialis*	K, Ca, Cr, Mn, Fe, Ni, Cu, Zn, Sr, Cd and Pb	[46]
39	Central Adriatic Sea, Italy. (7 sites; 2006–2007)	*Mytilus galloprovincialis*	Mn, Fe, Ni, Cu, Zn, Cd, Sn, Hg and Pb	[47]
40	Bay of Islands, northern New Zealand (4 sites; 2005)	*Perna canaliculus*	Cd, Hg, As, Pb and Sn	[48]
41	Seafood markets in Metro Manila, Philippines (3 sites; 2007)	*Perna viridis*	Cd, Cu, Pb and Zn	[49]
42	Bilbao estuary (Spain) (2002–2004)	Unspecified	Cd, Co, Cr, Cu, Hg, Mn, Ni, Pb, V and Zn	[50]
43	Cantabrian Coast, northwest Spain (10 sites; 2004–2006)	*Mytilus galloprovincialis*	As, Cd, Co, Cr, Cu, Ni, V, Hg, Se, Sn, Pb, Mn and Zn	[51]
44	Coastal waters of European continent (17 sites; 2002–2004)	*Mytilus edulis*	Fe, Mn, Pb, Zn, and Cu	[52]
45	Maule Region, Chile (3 sites; 2005–2006)	*Ameghinomya antiqua*, *Aulacomya atra* and *Mytilus chilensis*	Cd, Cr and Pb	[53]
46	Anzali wetland, Iran (2 sites; 2006)	*Anodonta cygnea*	Cd, Cu and Pb	[54]
47	Brown Bay (Beagle Channel), Argentina (1 site; 2007–2008)	*Mytilus edulis chilensis*	Cu, Zn, Fe, Cd and Pb	[55]
48	New Caledonia lagoon (2 sites; 2003)	*Gafrarium tumidum* and *Isognomon isognomon*	As, Cd, Co, Cr, Mn and Zn	[56]
49	Marmara Sea, Turkey (10 sites; 2009).	*Mytilus galloprovincialis*	Zn, Cu, Cd, Hg and Pb	[57]
50	Gulf of Finland (Baltic Sea) (3 sites; 2011)	*Mytilus trossulus*	As, Cd, Co, Cr, Cu, Ni, Pb, V and Zn	[58]
51	Gangetic delta, India (2 sites; 2010)	*Saccostrea cucullata* and *Crassostrea madrasensis*	Zn, Cu, Pb and Cd	[59]
52	Todos os Santos Bay, Bahia, Brazil (34 sites; 2006–2010)	*Anomalocardia brasiliana*, *Brachidontes exustus*, *Crassostrea rhizophorae, Mytella guyanensis.*	Al, As, Ba, Cd, Co, Cr, Cu, Fe, Hg, Mn, Pb, Se, Sr, V and Zn	[59]
53	Cape Town Harbour, South Africa (Unspecified; 2011)	*Mytilus galloprovincialis*	K, Ca, Fe, Cu, Zn, Si, Sr, Al, Mn, Pb, As, Hg, V, Cr, Sn, Cd, Ni and Co	[60]
54	The Gulf of Annaba, Algeria (4 sites; 2006–2007)	*Perna perna*	Cd, Cu, Cr, Fe, Hg, Mn, Ni, Pb and Zn	[61]
55	Pagassitikos Gulf (Aegean Sea, Eastern Mediterranean (6 sites; 2008)	Unspecified	Cd, Cu, Cr, Ni, Zn, Fe, Mn and Pb	[62]
56	The Straits of Johore, Malaysia (2 sites; 2009)	*Perna viridis*	Cd, Cu, Fe, Ni, Pb and Zn	[63]
57	Catania fish market, Italy (2012)	*Donax trunculus*	As, Cd, Cr, Pb, Mn, Ni, V and Zn	[64]
58	Baja California, Mexico (15 sites; 1995)	*Modiolus Capax*	Cd, Co, Cu, Fe, Mn, Ni, Pb and Zn	[65]
59	Nova Scotia, Canada (11 sites; 2008–2012)	*Mytilus edulis*	As, Cd, Cu, Hg, Pb and Zn	[66]
60	Libyan coast (16 sites; 2009)	*Mytilus galloprovincialis*	Hg, Cr, Pb, Cd, Cu, Zn and Ni	[67]
61	Boka Kotorska Bay, Adriatic Sea, Montenegro (7 sites; 2009)	*Mytilus galloprovincialis*	Fe, Mn, Cu, Zn, Co, Ni, Cd, Pb and Hg	[67]
62	The eastern Adriatic Coast, Croatia (13 sites; 2012–2013)	*Mytilus galloprovincialis*	As, Cd, Hg and Pb	[68]
63	Cape Peninsula, Cape Town, South Africa (5 sites; 1985–2008).	*Mytilus galloprovincialis*	Cu, Cd, Pb, Zn, Hg, Fe and Mn	[69]
64	Abu-Qir Bay, Alexandria, Egypt (1 site; 2013)	*Pinctada radiate* and *Paphia textile*	Cd, Cr, Cu, Fe, Mn, Ni, Pb and Zn	[70]
65	Kent, South-east England (4 sites; 2012)	*Mytilus edulis*; *Crassostrea gigas*	Cd, Cu, Pb and Zn	[71]
66	Four seas at Turkish coastline (20 sites; 2011)	*Mytilus galloprovincialis*	Ag, Al, As, Cd, Co, Cr, Cu, Fe, K, Mn, Ni, Pb, Sn, V and Zn	[72]
67	Ulsan and Onsan Bays, Korea	*Mytilus galloprovincialis*	Ag, As, Cd, Co, Cr, Cu, Fe, Hg, Mn, Ni, Pb, Sb, Se, Sn and Zn	[73]
68	Sürmene Bay, Black Sea, Turkey	*Mytilus galloprovincialis*	As, Co, Cr, Cu, Mn, Mo, Ni, Pb and Zn	[74]
69	Cala Iris offshore, Northern Morocco	*Mytilus galloprovincialis*	Cd, Cr, Cu, Fe, Ni, Zn, Co and Pb	[75]
70	Southwest of Buenos Aires Province (Bahía Blanca Estuary and Pehuen-Có beach), Argentina	*Brachidontes rodriguezii*	Cd, Cu, Pb, Zn, Ni and Cr	[76]
71	Marmara sea coast of Tekirdag, Turkey	*Mytilus galloprovincialis*	As, Cd, Cr, Cu, Ni, Zn and Pb	[77]
72	Gulf of Naples and Domitio littoral, Italy	*Mytilus galloprovincialis*	PCBs, dioxins, PAHs, Pb, Cd and Hg	[78]
73	Saldanha Bay, South Africa	*Mytilus galloprovincialis* and *Choromytilus meridionalis*	As, Cu, Cr, Fe, Zn, Cd and Pb	[79]
74	Sariçay Stream, Turkey	*Unio crassus*	Cd, Co, Cr, Cu, Fe, Hg, Mn, Ni, Pb, U and Zn	[80]
75	Urubuqueçaba Island, Santos Bay, Brazil	*Perna perna*	Al, Cd, Cr, Cu, Fe, Mn, Ni, Pb and Zn	[81]
76	North Sea and Baltic Sea	*Mytilus edulis*	Co, Ni, Cd, Cu, Pb and As	[82]
77	Safi areas in the northwestern Atlantic coast, Morocco	*Mytilus galloprovincialis*	Cd and Cu	[83]
78	Bohai Sea, Yellow Sea, East China Sea and South China Sea, China	*Mytilus edulis*, *Mytilus unguiculatus* and *Perna viridis*	Na, K, Ca, Mg, P, Ag, Cd, Cr, Cu, Ni, Pb, Ti and Zn	[84]
79	Limfjorden, Denmark	*Mytilus edulis* L.	Cd, Cu, Ni, Pb and Zn	[85]
80	Sydney Estuary, Australia	*Xenostrobus securis*	Cd, Cr, Cu, Pb and Zn	[86]
81	Coastal areas of Casablanca, Morocco	*Mytilus galloprovincialis*	Cu, Zn, Ni and Pb	[87]
82	San Jorge Gulf, Argentine (2010)	*Mytilus edulis platensis*	Al, Ag, As, B, Ba, Be, Cd, Cu, Co, Cr, Fe, Mn, Mo, Ni, Pb, Se, Sr, V and Zn	[88]
83	Ría de Arousa in NW Spain and Bizerte lagoon in N Tunisia	*Mytilus galloprovincialis*	Cu, Co, Pb, Cd, Cr, As and Ni	[89]
84	Harbor waters of Kristiansand, Norway	*Mytilus edulis* spp.	As, Cd, Cr, Cu, Hg, Ni, Pb and Zn	[90]
85	Port Phillip Bay, Victoria, Australia (2017 and 2018)	*Mytilus galloprovincialis*	Cd, Pb, Cu, Zn, Cr, Se, Hg and As	[91]
86	Marche Region coast, Central Adriatic Sea, Italy (2008–2018)	*Mytilus galloprovincialis* and *Chamelea gallina*	Pb, Cd, V, Ni, Cr and As	[92]
87	Keban Dam Reservoir, Turkey	*Unio elongatulus eucirrus*	Co, Cr, Cu, Cd, Mn, As, Fe, Pb and Zn	[93]
88	South African Harbours include Cape Town, Durban, East London, Mossel Bay, Port Elizabeth and Richards Bay Harbours	*Perna perna*	Al, As, Cd, Co, Cr, Cu, Fe, Mn, Mo, Ni, Pb, Se, Sr, U and Zn	[7]
89	Straits of Johore, Peninsular Malaysia	*Perna viridis*	Ag, As, Be, Co, Cr, Cs, Hg, li, Mn, Se, Sr and V	[94]
90	Straits of Johore, Peninsular Malaysia	*Perna viridis*	Ag, As, Be, Co, Cr, Cs, Hg, li, Mn, Se, Sr and V	[95]
91	Straits of Johore, Peninsular Malaysia	*Perna viridis*	Ag, As, Be, Co, Cr, Cs, Hg, li, Mn, Se, Sr and V	[96]
92	Kampung Pasir Puteh, Peninsular Malaysia	*Perna viridis*	Cu	[97]

**Table 2:** A review on the use of shells of bivalves in metal pollution studies from some of the available literature.

No.	Mussel Species	Metals Investigated	Studies Conducted and Major Findings	References
1	*Crassostrea virginica*	Cd, Ca, Cu, Fe, Mg, Mn, Sr and Zn.	This study confirmed the capacity of oysters to concentrate several elements in their valves as concentration of these elements increased in ambient sea water (3 sites; 1977).	[104]
2	*Mytilus edulis*	Cd, Cu, Zn, Pb, Ag, Ni and Pu.	Bivalve shells are advantageous in monitoring of heavy metal pollution because of their convenience in storage and handling. Shells are superior to soft tissues in terms of the sensitivity towards metal levels in the environmental over the long term.	[105]
3	*Mytilus edulis*	Si, Ca, Fe, Cu and Sr	Accumulation and concentration of Cu in the organic periostracum suggest that *Mytilus* shell may also prove useful as a monitor of metallic element pollution.	[106]
4	*Mytilus edulis*	Fe, Mn, Ni, Pb, Cu, Co, Zn, Cd, Ca and Mg	Shells contain higher concentrations of Fe, Mn, Ni, Pb and Ca in comparison to the soft tissues denoting high bioaccumulation capacity and the potential of shells as biomonitoring materials (1981; Gdansk and Puck Bay, Poland).	[107]
5	*Mya truncate*	Pb, Zn, Cu, V, Ni, Cu and Co	This study suggested that shells of bivalves may be an essential and underutilized assessment tool for pollutant assessments in the environment (3 sites near Pangnirtung, Northwest Territories; 1985).	[108]
6	*Crassostrea virginica*	Cd, Cr, Cu, Fe, Mn, Pb and Zn	Cd is enriched in oyster shells. Variations of metal concentrations in different parts of shells can record environmental changes during oyster growth (1986; US Gulf of Mexico Bay).	[109]
7	*Modiolus modiolus*	Cu, Zn and Pb	This study supported the use of shells as historical archives for heavy metals levels in the marine environment (1984 from 2 sites in the southern North Sea)	[110]
8	*Mytilus edulis trossulus*	Zn, Mn, Cu and Fe	Southern Baltic, Poland (23 sites; 1997). Variations of the 4 metals were recorded among the three regions, with Mn being higher in the shells in comparison with soft tissues.	[28]
9	*Perna viridis*	Heavy metals	High occurrence of shell deformities observed in certain sites could be attributed to heavy metal pollution in the west coast of Peninsular Malaysia.	[111]
10	*Perna viridis*	Cd, Pb and Zn	Field collected and laboratory experimental mussels. The findings of this study recommended the total shell of *P. viridis* as a potential biomonitoring material for long-term contamination of Cd, Pb and Zn (1998–2001; 12 sites from the west coast of Peninsular Malaysia).	[31]
11	*Perna viridis*	Cu, Co, Ni, Cd, Zn, Mn, Cr, Fe and Pb	Hong Kong (2 sites; unspecified). Higher levels of Cu, Zn, Mn and Fe in the shells of mussels collected from contaminated Kennedy Town site within Victoria Harbour than uncontaminated Kat O site.	[32]
12	*Perna viridis*	Zn	Wide range of Zn accumulation and close positive correlation with the shells indicated that the shells of *P. viridis* is a biomonitoring material for Zn.	[112]
13	*Mercenaria Mercenaria*	Pb and Ca	This study recommended the possibility of revealing large and long-term changes in the environmental Pb concentrations if sufficient specimens are pooled together for analysis (1949–2002; North Carolina, USA).	[113]
14	*Pleurobema oviforme*	Hg	The study of shell-based monitoring means there is no need of live samples and thus make ways for more standard strategies to be applied to environmental monitoring. This strategy is especially useful if there is no prior knowledge on the extirpation and pollution histories of the study area.	[114]
15	hydrothermal vent bivalve *Bathymodiolus azoricus*	Fe, Cu and Zn	Shells are good indicators of environmental levels of Fe, Cu and Zn at hydrothermal vents and thus may be considered markers of putative changes in metal exposure over the mussel’s lifespan.	[115]
16	*Perna viridis*	Cd, Cu, Pb and Zn	The findings based on stepwise regression analysis showed that the transport of Cd, Pb and Zn into the mussel shells could have caused the shell deformities.	[116]
17	*Ensis siliqua*	Zn, Cd, Pb, U, Ba, Sr and Mg	Consistent regional distribution of metals was found in this study in which the sources of pollution and patterns of seawater migration are known (1990s; 13 locations around the west coast of mainland Britain).	[117]
18	*Perna perna*	Cr, Mn, Ni, Cu, Zn, Cd and Pb	Aquarium experiments; confirm the use of the mussel *Perna perna* as a good biomonitoring material for toxic elements based on the new formation of growth rings on the mussels’ shells which corresponded with the increase in most pollutants at the study site.	[118]
19	*Mytilus edulis*	Hg, Pb, Cd, Cu, Zn, Cr, Ni, Fe, Mn, V, Li and Al	This study supported the suitability of mussel shells as biomonitoring surveys in the Poland coast of Baltic. (2005; field collected from 12 sites on the Polish coast of Baltic Sea)	[119]
20	*Elliptio complanata*	Mn, Cu, Sr and Ba	The factors affecting the content of metals of different shell layers in bivalves will assist the understanding of potential relationships between the chemistry of ambient fluids in freshwater environments and shell carbonate over the incremental growth history of the shell. This relationship is indispensable for the use of trace element concentrations as paleoenvironmental proxies (2003; 4 streams in South Carolina).	[120]
21	*Mytilus galloprovincialis*	K, Ca, Cr, Mn, Fe, Ni, Cu, Zn, Sr, Cd and Pb	Ca, Cu, Sr, and Ba were detected in the shell of mussels where the metal accumulation reveals the duration of exposure and the levels of pollution. Shells could serve as essential environmental metal concentrations records (Eastern Black Sea, Turkey (5 sites; unspecified)).	[46]
22	*Anadara granosa*	Cd, Cu, Fe, Ni and Zn	Studied heavy metals in the cockle shells from three sites in coastal areas of Peninsular Malaysia.	[121]
23	*Perna viridis*	Cd, Cu, Ni, Fe and Zn	Studied heavy metals in the mussel shells from two sites of northern part of Peninsular Malaysia.	[122]
24	*Psammotaea elongata*	Cd, Cu, Pb and Zn	Studied heavy metals in the bivalve shells from one site in Kelantan, Peninsular Malaysia.	[123]
25	*Pholas orientalis*	Cd, Cu, Ni, Pb, Fe and Zn	Studied heavy metals in the bivalve shells from two sites in Selangor coastal areas, Peninsular Malaysia.	[124]
26	*Perna viridis*	Cd and Pb	Suggested the potential of periostracum of *P. viridis* as a biomonitor for Pb but not for Cd. However, further studies should be conducted to prove the potential of periostracum as a good biomonitoring tissue for heavy metal pollution in tropical coastal waters.	[125]
27	*Perna viridis*	Cu and Zn	Periostracum is suggested as a good biomonitoring tissue for Cu, but not for Zn, based on the higher levels of Cu in periostracum in comparison to soft tissues and closer relationship of the Cu between periostracum and sediment.	[103]
28	*Bathymodiolus* mussels and *Calyptogena magnifica*, *Archivesica gigas*, and *Nuculana grasslei* clams	Fe, Mn, Zn, Cu, Cd, Pb, Ag, Ni, Cr, Co, As, Se, Sb and Hg	Enriched metals (Fe and Mn) were found in bivalve shells from hydrothermal fields with black smokers. It was also evident that in the early ontogeny of the shells essential metals such as Fe, Mn, Ni, and Cu were more actively accumulated. The shells of the bivalve displayed efficient accumulation functions due to high concentration factors of majority of the metals (seven hydrothermal vent fields of the Mid-Atlantic Ridge).	[126]
29	*Mytilus galloprovincialis*	K, Ca, Fe, Cu, Zn, Si, Sr, Al, Mn, Pb, As, Hg, V, Cr, Sn, Cd, Ni and Co	The study showed higher metal levels in the soft tissues in comparison to shells but shells might also give relevant information on the environmental metal pollution status. Two visible patterns of bioaccumulation in soft tissues (As, Cd, Hg, Pb and Sn) and shells (Co, Cr, Mn, Ni, Pb and V) were also found, indicating strong associations, most likely of anthropogenic origin (Cape Town Harbour, South Africa, 2011).	[60]
30	*Perna viridis*	Ag, As, Be, Co, Cr, Cs, Hg, Li, Mn, Se, Sr and V	It is difficult to explain the outcome of this study as all metal data on soft tissues and shells presented were after the transplantation periods from a polluted site to two unpolluted sites in the Straits of Johore.	[127]
31	*Unio tumidus*	Zn, Cu, Fe, Pb, Ni and Cd	These results reflected contemporary anthropogenic pollution of the environment with heavy metals and confirm the possibility of using the shells in the assessment of heavy metal pollution levels (Szczecin Lagoon, SW Baltic basin).	[6]
32	*Unio crassus, Unio pictorum, Unio tumidus*	Ca, Cd, Cr, Cu, Fe, Hg, Ni, Pb and Zn	The results indicated that metal transfer between mussel shells and surrounding deposits does not occur. They suggested that the shells could be successfully used as independent bioindicators.	[128]
33	*Bathymodiolus platifrons*	Ca, K, Mg, Sr, Ag, Al, As, Ba, Cd, Co, Cr, Cu, Li, Fe, Mn, Mo, Ni, Pb, V and Zn	Concentrations of metals were highest in the new-growth outer edges of shells in comparison to older shell material, which suggests that trace metals have become more abundant in the ambient seawater in recent years (a cold seep at the northern continental slope of the South China Sea).	[129]
34	*Elliptio dilatata and Elliptio complanata*	Wastewater metals	They found that freshwater mussel shells may be used to monitor changes in water chemistry through time and help identify specific pollutant sources (Pennsylvania, USA).	[130]
35	*Mytilus galloprovincialis*	As, Ba, Cd, Co, Cr, Cu, Fe, Hg, Ni, Pb, V, Sr, Zn and Mn	A decrease in the concentration of most elements in their shells with an increase in the age of the organism with the exception of V, Sr and Fe (Inal Bay, the Black Sea).	[131]
36	*Perna viridis*	Hg, Pb, Cd, Cr, and Sn	The main cause of malformations in green mussels was suspected to be Pb, Hg and Sn (Jakarta Bay, Indonesia).	[132]

**Table 3:** A review on the use of bivalves’ biomarkers in metal pollution studies on bivalves from some of the available literature.

No.	Mussel Species	Metals Investigated	Biomarkers Used	References
1	*Perumytilus purpuratus*	Cu under laboratory conditions.	Lysosomal stability in hemocytes and the degree of vacuolization and the content of lipofuscin granules in digestive cells.	[141]
2	*Perna viridis*	Metals, organochlorines, polycyclic aromatic hydrocarbons and organotins	Molecular (DNA strand breaks, DNA adducts, micronuclei, enzyme antioxidants and metallothionein), cytological (lysosomal membrane stability, phagocytosis), morphological (gill damage, physiological (heart rate, clearance rate, scope for growth and condition index).	[99]
3	*Dreissena polymorpha*	Cr, Ni, Cu, Zn, As, Cd, Pb and Hg	Levels of metallothioneins, activities of ethoxyresorufin-O-deethylase, oxidative stress biomarkers (glutathione content, enzymatic activities of superoxide dismutase, catalase, glutathione S-transferase, glutathione peroxidise and glutathione reductase), levels of lipid peroxidation and DNA strand breaks.	[142]
4	*Mytilus galloprovincialis*	Cu, Ni, Fe and Zn	Integrated biomarker response (metallothioneins, glutathione S-transferase, catalase, acetylcholinesterase and RNA:DNA ratio).	[62]
5	*Mytilus edulis*	Cu	DNA strand breaks, levels of glutathione, histopathological changes, and clearance rate	[143]
6	*Mytilus galloprovincialis*	Fe, Zn, Cu, Ni, Cr, Cd and Pb:	Lysosomal membrane stability and histopathology of gonad and digestive gland.	[140]
7	*Mytilus galloprovincialis*	Cu, Ni, Pb, Cr, Cd, Fe and Zn	Intralysosomal metal levels in digestive cells, metallothionein content in digestive gland tissue, peroxisome proliferation, the exposure component of the bell-shaped changes in digestive gland AOX activity, intracellular accumulation of neutral lipids in digestive gland diverticula; ALP level in mantle (gonad) of male mussels. Genotoxicity biomarkers: MN frequency measured in haemocytes. Oxidative stress biomarkers: MDA levels in digestive gland and LPF accumulation in digestive cells. General stress biomarkers: Lysosomal membrane labilisation period in digestive cells; cell-type composition of digestive tubule epithelium. Population fitness biomarker: accumulated mortality in air exposed mussels against exposure time (days) and LT50 (days).	[144]
8	*Mytilus* sp.	As, Cd, Co, Cr, Cu, Hg, Methyl-Hg, Mn, Ni, Pb and Zn	Haemocyte lysosomal stability, frequency of irregular nuclei in haemocytes, and frequency of micronuclei in haemocytes	[145]
9.	*Mytilus galloprovincialis*	Pollutant stress.	Antioxidant enzymes (catalase and glutathione peroxidase, a phase II detoxification enzyme (glutathione S-transferase) and a neurotransmitter catabolism enzyme (acetylcholinesterase)	[146]
10.	*Aulacomya atra atra*	Fe, Al, Zn, Cu, Cd and Pb	Reactive oxygen species, lipid radicals, malondialdehyde, superoxide dismutase, catalase, glutathione S-transferase and metallothionein.	[147]
11	*Mytilus galloprovincialis*	Cd, Cr, Cu, Fe, Ni, Pb and Zn	Lysosomal membrane stability and lysosomal structural changes and changes in cell-type composition in digestive gland epithelium	[148]
12	*Mytilus galloprovincialis*	As, Cd, Co, Cr, Cu, Hg, Mn, Ni, Pb, V and Zn	Condition index, phospholipids, total and neutral lipids.	[149]
13	*Mytilus* spp.	Al, As, Ba, Cd, Co, Cu, Fe, Ni, Pb and Zn	Stress on stress, condition index, cellular energy allocation, micronuclei formation, lysosomal membrane stability, basophilic cell volume and neutral lipid accumulation.	[150]
14	*Mytilus galloprovincialis*	Hg, Cd, Pb, Cu, Zn and As	Antioxidant enzymatic activities, lipid peroxidation, and the physiological rates integrated in the scope for growth biomarker (clearance rate), biological variables (shell thickness), condition index, gill index, gonado somatic index, hepato somatic index, total reproductive potential, sexual maturity index.	[151]
15	*Perna canaliculus*	As, Cd, Cu, Pb, Ni and Zn	Physiological biomarkers (clearance rate, absorption efficiency, respiration rate, excretion rate and oxygen to nitrogen ratio, scope for growth, condition index), biochemical biomarkers (metallothionein-like protein content, catalase activity and alkaline phosphatase activity, lipid peroxidation levels), immunocytotoxic and cytogenotoxic biomarkers (haemocyte count, nuclear aberrations).	[152]
16	*Mytilus galloprovincialis*	Pb, Zn, Ni, As, Hg, Cr, Cu and Cd	Condition index, stress on stress, micronuclei frequency, lysosomal membrane stability test, neutral lipids content, lysosome-to-cytoplasm ratio, lipofuscin content, oxidative stress (catalase activity, malondialdehyde content and protein carbonyl derivates), vitellogenin-like proteins, and metallothionein content.	[153]
17	*Mytilus galloprovincialis*	Cu, Zn, Pb, Cd	Catalase (CAT), glutathione s-transferase (GST), and condition indices.	[154]
18	*Margaritifera margaritifera*	Cu, Cr, Zn, Cd and Ni	Transcriptomic responses; Cr, Zn, Cd, and Ni were the main factors correlated with transcription levels, with effects on translation, apoptosis, immune response, response to stimulus, and transport pathways.	[155]
19	*Dreissena polymorpha*	Platinum	Metal-associated biomarker responses; glutathione-S-transferase (GST) and catalase (CAT) activity, lipid peroxidation and metallothionein (MT) induction.	[156]
20	*Mytilus galloprovincialis*	Pb, Cd and Cu	Tissue distribution; the metals concentrated in the digestive gland, although the percentages of each element varied between compartments and varied between tissues according to the treatment.	[157]
21	*Mytilus galloprovincialis*	Pb, Cu and Zn	Biological indices such as biometric and physiological indices.	[158]
22	*Perna perna*	As, Cd, Ni and Se	Gill metallothionein (MT), reduced glutathione (GSH), carboxylesterase (CarbE) and lipid peroxidation.	[159]
23	*Mytilus galloprovincialis*	Stable isotopes and metal(loid	Condition indices.	[160]
24	*Dreissena polymorpha*	Cd, Cu, Pb, Ni and Zn	Cytochrome-c-oxidase–cox, and ATP synthase–atp, metallothionein, glutathion-s-transferase, catalase, superoxyde dismutase, glutathion peroxidase, amylase and cellulase.	[161]
25	*Anodonta cygnea*	Fe, Zn, Mn, Pb, Cu, Cr, Ni and Cd	DNA damage.	[162]
26	*Mytilus galloprovincialis*	Cu, Cd and Hg	Oxidative—damage of protein expression and modification—damage on the protein synthesis machine integrity and specifically on translation factors and ribosomal proteins expression and modifications.	[163]
27	*Mytilus galloprovincialis*	Cu, Cd and Hg	Oxidative damage of 18S and 5S ribosomal RNA in digestive gland; structural changes, such as base modifications, scissions, and conformational changes, caused in 18S and 5S ribosomal RNA (rRNA).	[164]
28	*Mytilus galloprovincialis*	Chlorpyrifos (CHP), Benzo(a)pyrene (B(a)P), Cd and Cu	Variations of AChE, MTs, CAT and LPO variations responses.	[165]
29	*Pyganodon grandis*	Be, Pb, Al, V, Cr, Co, Ni, Mo and Ni.	Metallothionein levels and oxidative stress.	[166]
30	*Aulacomya atra*	Al, Cr, Cu, Mn, Ni and Zn	Glutathione, superoxide dismutase, glutathione-S-transferase, reactive oxygen species and total oxyradical scavenging capacity.	[167]
31	*Mytilus galloprovincialis*	^110m^Ag and ^109^Cd	Tissue distribution, filtration rate, haemocyte viability and lysosomal membrane stabilization.	[168]
32	*Perna viridis*	Cd, Cu and Zn	Biomarker tests, including neutral red retention time test (NRRT) and micronuclei (MN) test.	[169]
33	*Anodonta anatina*	Pb, Cr and Cu	DNA damage in gills.	[170]
34	*Dreissena polymorpha*	Ni and Pb	Lysosomal membrane stability and respiration rate; lysosomal membrane stability in haemocytes of the invasive mollusk zebra mussel; changes in the respiration rate and survival under acute heavy metal exposure.	[171]
35	*Unio mancus*	Cd, Cu, Pb, Zn and Ni	Metallothionein level, reduced GSH level, MDA level.	[172]
36	*Xenostrobus securis*	Cd, Cr, Cu, Pb and Zn	Genotoxic (DNA damage, via the micronucleus frequency test) and cytotoxic (lysosomal membrane stability (cellular integrity).	[173]
37	*Mytilus galloprovincialis*	Cd, Cu, Pb and Zn	Mussel gills in metal pollution biomonitoring is a promising tool for the detection of changes in bioavailable metals in the environment, especially for essential metals such as Cu and Zn.	[174]

**Table 4:** A review on the human health risk assessments of heavy metals in bivalves from some of the available literature.

No.	Mussel Species	Metals Investigated; Findings	Locations/Country	References
1	*Tapes decussatus*; *Mytilus galloprovincialis*	Hg, Cd, Pb, Cr, Zn and Cu; Total hazard index (THI) values were greater than one in both bivalves, having a potential risk for consumers.	Homa Lagoon, Eastern Aegean Sea	[178]
2	*Cristaria plicata*	Zn, Pb, Cd, As, Cu and Cr; the hazard index (HI) values for adults and juveniles were higher than 1, suggesting significant risks of noncarcinogenic effects to humans by exposure to multiple metals.	Dongting Lake, China	[179]
3	*Mytilus galloprovincialis*	Pb, Cd, Cr, Ni, Co, Cu, Zn, Mn and Fe; the Cr measured in mussels was considered “extreme”, according to the consumption rate limit for mussels which limits their consumption to 0.5 kg/day.	Algerian coast	[180]
4	*Mytilus galloprovincialis*, *Mytilus edulis*, *Mytilus chilensis*, *Venerupis philippinarum*, *Perna canaliculus*, *Tapes decussatus*, *Tapes semidecussatus*, *Meretrix meretrix*, *Meretrix lyrata*	Cd, Pb, Hg, As, Cr, and Ni; the average Italian consumption of molluscs did not pose a risk for consumers, except Ni.	Italian market	[181]
5	*Perna viridis*	Pb, Cd, Cu and total Hg; the values of calculated target hazard quotient and hazard index for Pb and Cd were >1.0.	Kampung Pasir Puteh, Peninsular Malaysia	[182]
6	*Mytilus galloprovincialis*	As, Cd, Hg, Cu, Cr, Mn, Fe, Ni, Zn and Pb; THQ values for the toxic and essential metals were <1.0.	Black sea, Bulgaria	[183]
7	*Mytilus galloprovincialis*	Cu, Zn, Mn and Fe. THQ values for all tested trace metals were <1.0.	Strait of Canakkale, Turkey	[184]
8	*Crassostrea palmula*; *Mytella strigata*	Cd, Cr, Cu, Co, Zn, Mn, Ni, Pb, Hg, Fe and U. Pb in oysters exceeded legal limits set for bivalve mollusks in EU.	Estero de Urias lagoon, Gulf of California, USA.	[185]
9	*Perna viridis*	Cd, Pb, Cu and Zn; Pb-contaminated green mussels with HQ values > 1.0.	Semarang coastal waters, Central Java, Indonesia	[186]
10	*Mytilus galloprovincialis*	Cd, Pb and Hg. All THQ and HI values were <1.0.	Varna Bay of Black Sea, Bulgaria	[187]
11	*Brachidontes rodriguezii*	Cd, Cu, Pb, Mn and Fe; the metal contents in mussels met the national and international standards for safe consumption.	Bahía Blanca Estuary (Argentina),	[188]
12	*Perna viridis*	Cd, Cu, Fe, Pb Ni and Zn. THQ values were <1.0 for average level mussel consumers but higher than 1 for high level mussel consumers in some sites.	Malaysia	[189]

## Data Availability

Not applicable.
